# Anti-tumor activity of olaparib, a poly (ADP-ribose) polymerase (PARP) inhibitor, in cultured endometrial carcinoma cells

**DOI:** 10.1186/1471-2407-14-179

**Published:** 2014-03-13

**Authors:** Aki Miyasaka, Katsutoshi Oda, Yuji Ikeda, Osamu Wada-Hiraike, Tomoko Kashiyama, Atsushi Enomoto, Noriko Hosoya, Takahiro Koso, Tomohiko Fukuda, Kanako Inaba, Kenbun Sone, Yuriko Uehara, Reiko Kurikawa, Kazunori Nagasaka, Yoko Matsumoto, Takahide Arimoto, Shunsuke Nakagawa, Hiroyuki Kuramoto, Kiyoshi Miyagawa, Tetsu Yano, Kei Kawana, Yutaka Osuga, Tomoyuki Fujii

**Affiliations:** 1 Department of Obstetrics and Gynecology, Faculty of Medicine, The University of Tokyo, 7-3-1 Hongo Bunkyo-ku, Tokyo 113-8655, Japan; 2 Laboratory of Molecular Radiology, Center for Disease Biology and Integrative Medicine, Graduate School of Medicine, The University of Tokyo, Tokyo, Japan; 3 Department of Obstetrics and Gynecology, Faculty of Medicine, Teikyo University, Tokyo, Japan; 4 Kanagawa Health Service Association, Kanagawa, Japan; 5 Department of Obstetrics and Gynecology, National Center for Global Health and Medicine, Tokyo, Japan

**Keywords:** PARP inhibitor, Homologous recombination, Endometrial cancer, PTEN, RAD51

## Abstract

**Background:**

PTEN inactivation is the most frequent genetic aberration in endometrial cancer. One of the phosphatase-independent roles of PTEN is associated with homologous recombination (HR) in nucleus. Poly (ADP-ribose) polymerase (PARP) plays key roles in the repair of DNA single-strand breaks, and a PARP inhibitor induces synthetic lethality in cancer cells with HR deficiency. We examined the anti-tumor activity of olaparib, a PARP inhibitor, and its correlation between the sensitivity and status of PTEN in endometrial cancer cell lines.

**Methods:**

The response to olaparib was evaluated using a clonogenic assay with SF50 values (concentration to inhibit cell survival to 50%) in 16 endometrial cancer cell lines. The effects of PTEN on the sensitivity to olaparib and ionizing radiation (IR) exposure were compared between parental HEC-6 (PTEN-null) and HEC-6 PTEN + (stably expressing wild-type PTEN) cells by clonogenic assay, foci formation of RAD51 and γH2AX, and induction of cleaved PARP. The effects of siRNA to *PTEN* were analyzed in cells with wild-type PTEN.

**Results:**

The SF50 values were 100 nM or less in four (25%: sensitive) cell lines; whereas, SF50 values were 1,000 nM or more in four (25%: resistant) cell lines. PTEN mutations were not associated with sensitivity to olaparib (Mutant [n = 12]: 746 ± 838 nM; Wild-type [n = 4]: 215 ± 85 nM, p = 0.26 by Student’s *t* test). RAD51 expression was observed broadly and was not associated with PTEN status in the 16 cell lines. The number of colonies in the clonogenic assay, the foci formation of RAD51 and γH2AX, and the induction of apoptosis were not affected by PTEN introduction in the HEC-6 PTEN + cells. The expression level of nuclear PTEN was not elevated within 24 h following IR in the HEC-6-PTEN + cells. In addition, knocking down PTEN by siRNA did not alter the sensitivity to olaparib in 2 cell lines with wild-type PTEN.

**Conclusions:**

Our results suggest that olaparib, a PARP inhibitor, is effective on certain endometrial cancer cell lines. Inactivation of PTEN might not affect the DNA repair function. Predictive biomarkers are warranted to utilize olaparib in endometrial cancer.

## Background

Homologous recombination (HR) is a critical step for DNA repair, and certain types of cancers are HR defective, including BRCA1/2 deficiency [1,2]. Poly (ADP-ribose) polymerase (PARP) plays a key role in the repair of DNA single-strand breaks (SSBs) [1], and PARP inhibition leads to the accumulation of SSBs, which results in the development of DNA double strand breaks (DSBs) via the collapse of replication forks [3-5]. Tumor cells lacking functional BRCA1 and BRCA2 are deficient in the repair of DSBs by RAD51-mediated HR, which leads to cell cycle arrest and/or cell death [3]. Thus, targeting the HR defect, which is specific to cancer cells, and causing synthetic lethality by a PARP inhibitor is expected to be a promising therapeutic strategy in selected tumors [2]. Indeed, a PARP inhibitor, olaparib (AZD2281/ KU0059436), showed antitumor activity in cancer patients, especially with the *BRCA 1*/*2* mutations in breast and ovarian cancers [6,7]. However, BRCA status alone is not necessarily the only predictive biomarker for effective olaparib treatment because various types of genes are known to be involved in the HR process, including *PTEN*, *ATM*, *RAD51*[8-10]. Therefore, PARP inhibition might be useful for various types of tumors with HR defects, independent of the BRCA status (BRCAness).

Endometrial cancer is the fourth most common malignancy among women in the United States [11]. In endometrial cancer, the constitutive activation of the phosphatidylinositol 3-kinase (PI3K) pathway is induced by various types of alternations, including frequent mutations of *K*-*Ras* (10–20%), *PIK3CA* (25–36%), *AKT* (2%), and *PTEN* (34–56%) [12-15]. Additionally, the loss of heterozygosity (30–40%) of the *PTEN* locus at chromosome 10q23.31 is also associated with the inactivation of PTEN [16-18].

In addition to a negative regulator of the PI3K/AKT signaling pathway, PTEN contributes to maintaining genomic stability and DNA repair processes by regulating the expression of RAD51, a key protein in HR DNA repair [19]. The lack of PTEN also impairs CHK1 function, which results in the accumulation of DNA DSBs [20,21].

Dedes and coworkers showed that PTEN-deficient endometrial cell lines, which fail to elicit RAD51 to DNA damage sites, are sensitive to PARP inhibitors [3]. However, the correlation between PTEN status and RAD51 expression remains a debatable matter. For example, a recent study showed that PTEN deletion is not associated with the loss of RAD51 in prostate cancer cells [22].

The purpose of this study is to clarify the anti-tumor effect of olaparib on a panel of endometrial cancer cell lines and to assess the association among PTEN status, HR repair, and sensitivity to olaparib in endometrial cancer cells.

## Methods

### Cell lines and reagents

We used 16 endometrial cancer cell lines (Table 1). HHUA was purchased from RIKEN Cell Bank (Tsukuba, Japan). AN3CA, KLE, HEC-1B and RL95-2 were purchased from American Type Culture Collection (Manassas, VA). Ishikawa3-H-12 was a generous gift from Dr. Masato Nishida (National Hospital Organization Kasumigaura Medical Center, Japan). The other 10 cell lines were established by Hiroyuki Kuramoto [23].

**Table 1:** PTEN status in endometrial cancer cell lines

		**PTEN status**		
**Histological subtypes**	**Cell lines**	**Codon**	**Mutation**	**Predictive effect**
**Endometrioid adenocacioma**	HEC-6	INTRON 4 (+2)	T TO C	Splice donor
		289	1bp (A) del	Frameshift
	HEC-59	41	TAC to TAC	Tyr (Y) to His (H)
		233	CGA to TGA	Stop
		246	CCG to CTG	Pro (P) to Leu (L)
		267	1bp (A) del	Frameshift
	HEC-88	130	CGA to GGA	Arg (R) to Gly (G)
		173	CGC to TGC	Arg (R) to Cys (C)
		310	GAT to TAT	Asp (D) to Tyr (Y)
		341	TTT to TGT	Phe (F) to Cys (C)
	HEC-108	6	2bp (AA) del	Frameshift
		289	1bp (A) del	Frameshift
	HEC-116	Intron 2 (-1)	G to A	Splice acceptor
		173	CGC to TGC	Arg (R) to Cys (C)
		233	CGA to TGA	Stop
	HEC-151	33	3bp (ATT) del	In frame deletion
		76	2bp (AT) del	Frameshift
	HHUA	164	1bp (A) del	Frameshift
		289	1bp (A) del	Frameshift
	AN3CA	130	1bp (G) del	Nonsence
	Ishikawa3-H-12	289	1bp (A) del	Frameshift
		317-318	4bp (ACTT) del	Frameshift
	RL95-2	322	1bp (A) del and 1bp (A) ins	Frameshift
	HEC-251	10	AGC to AAC	Ser (S) to Asn (N)
	HEC-265	319	1bp (A) ins	Frameshift
	KLE	WT	None	
	HEC-1B	WT	None	
	HEC-50B	WT	None	
**Serous adenocarcinoma**	HEC-180	WT	None	

Histologically, only the HEC-180 cell line was classified as a serous adenocarcinoma; the other cell lines were classified as endometrioid adenocarcinomas. The culture conditions of the 13 endometrial cancer cell lines were described previously [13]. HEC-180, HEC-251, and HEC-265 cells were maintained in Eagle’s MEM with 10% FBS. HEC-6 cells stably expressing wild-type PTEN were generated by a retroviral infection, as described previously [13]. Phoenix cells were transfected with retroviral vectors (pFB-neo) that contained tandem affinity purification (TAP)-tagged wild-type PTEN using Lipofectamine 2000 (Invitrogen, Carlsbad, CA) and the resulting supernatants were used to infect HEC-6 cells. Drug selection was used to purify cell populations after infections by neomycin (500 μg/mL, 7 days).

Olaparib (AZD2281/KU0059436) was provided by AstraZeneca (London, UK). Olaparib was solved in DMSO, and the concentration of DMSO in each assay was 0.1%.

### Gene silencing and transient transfection

Cells were plated at approximately 30% confluence in 100-mm plates and incubated for 24 h before transfection with small interfering RNA (siRNA) duplexes at the concentrations indicated, using Lipofectamine 2000 RNAiMAX (Invitrogen, Carlsbad, CA) and Opti-MEM medium (Life Technologies, Grand Island, NY). The target sequence of siRNA specific for PTEN was described previously [12]. A negative control kit was used as a control (Invitrogen, Carlsbad, CA). HA-tagged wild-type PTEN expression plasmid was generated and transfected into PTEN mutant cell lines using Effectene transfection reagent (Qiagen, Valencia, CA, USA). HA-tagged pcDNA plasmid was used as a control.

### PCR and direct sequencing

The mutational status of PTEN (exons 1–9) was analyzed by PCR and direct sequencing as described previously [12]. The mutational status in 13 of the 16 endometrial cell lines and the PCR primers have been described previously [12,24]. The mutational status in the remaining three cell lines (HEC-180, HEC-251, and HEC-265) is presented in Table 1.

### Western blotting

Cells were lysed as described previously [12,25]. Antibodies specific for PTEN (138G6), phospho-PTEN (Ser^380^), AKT (Cell Signaling Technology), phospho-AKT (Ser^473^), PARP, cleaved PARP (Cell Signaling Technology, Beverly, MA), RAD51 (Millipore, MA, USA and Santa Cruz Biotechnology, CA, USA), and β-actin (Sigma-Aldrich, MO, USA) were used for western blotting, as recommended by the manufacturer. Proteins were visualized using an ECL western blot detection kit (GE Healthcare, Little Chalfont, UK).

### Immunofluorescence imaging

Immunocytochemistry was performed as described previously [26]. Primary antibodies to RAD51 (Millipore, MA, USA) (1:500 dilution) and γH2AX (Millipore, MA, USA) (1:500 dilution) and secondary antibodies to Alexa Fluor 488-conjugated chicken anti-mouse IgG and Alexa Fluor 568-conjugated goat anti-rabbit IgG (Invitrogen, Carlsbad, CA) (1:100 dilution) were used for analysis. Nuclei were visualized by staining with DAPI. The slides were briefly counterstained and analyzed by confocal fluorescence microscopy (Carl-Zeiss MicroImaging Inc., Oberkochen, Germany). The number of RAD51- and γH2AX-foci was evaluated in a mean of 100 cells.

### Cell cycle analysis

Cell cycle analysis was performed by flow cytometry, as previously described [24]. The cells were exposed to olaparib (10 μM) for the indicated time or were irradiated with 10 Gy after 24 h of irradiation. The cell cycle distribution was analyzed using CELL Quest pro ver. 3.1. (Beckman Coulter Epics XL Brea, CA). All experiments were repeated three times.

### Clonogenic assay

Cells were seeded in six-well plates at a concentration of 2,000 cells per well with olaparib (10 nM to 100 μM) or IR (2 Gy to 6 Gy). Cells were continuously exposed to olaparib with media during the incubation. After 14–21 days of incubation, the cells were fixed with methanol and stained with Giemsa (Wako). All experiments were repeated three times and the SF50 (surviving fractions at 50%) values, which indicate the concentration required to inhibit cell survival to 50%, were calculated by proliferation curves.

### IR

Cells were irradiated using a Shimadzu PANTAK HF-350 X-ray generator (1.0 mm Al +0.5 mm Cu filter; 200 kVp; 20 mA; Shimadzu, Kyoto, Japan).

### Statistical analysis

Data are expressed as the means ± standard deviations of three independent determinations. The significance of differences between the two samples was analyzed using Student’s *t*-test, and a p-value of <0.05 was considered to denote a statistically significant difference.

## Results

### RAD51 protein is expressed regardless of *PTEN* mutation status in endometrial cancer cell lines

**Figure 1:**
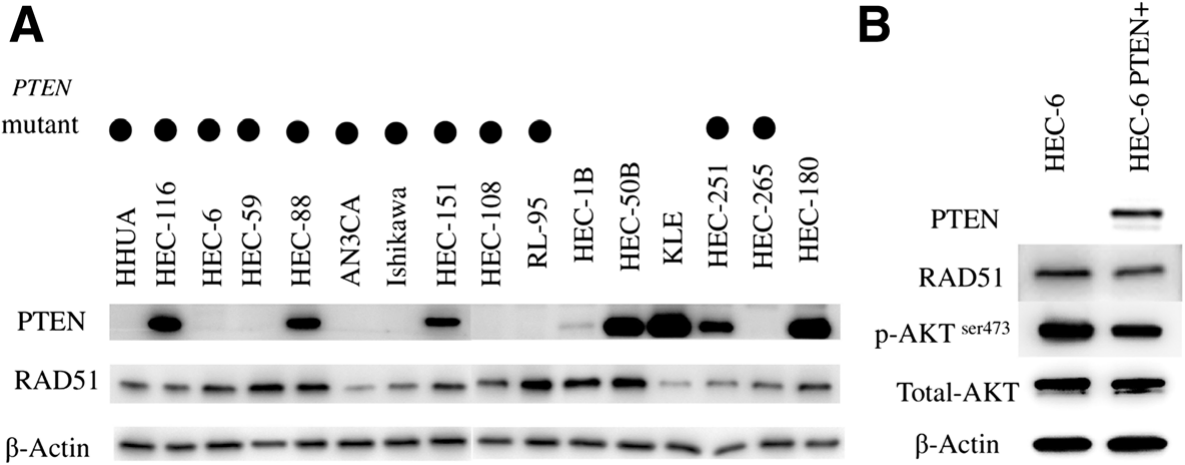
**Correlation between PTEN status and RAD51 expression in endometrial cancer cell lines. (A)** PTEN and RAD51 expression (western blot) in a panel of 16 endometrial cancer cell lines. Cell lines with a PTEN mutation are denoted as (●). **(B)** Establishment of the HEC-6-PTEN + cell line. Levels of PTEN, total/phosphorylated AKT, and RAD51 were evaluated by western blot analysis.

*PTEN* mutations were detected in 12 of the 16 (75%) endometrial cancer cell lines (Table 1). *PTEN* mutations were not observed in four cell lines (HEC-1B, HEC-50B, KLE, and HEC-180). Among the 12 cell lines with *PTEN* mutations, the expression of PTEN was observed in the HEC-116, HEC-88, HEC-151, and HEC-251 cell lines (Figure 1A). RAD51 expression was detected in all 16 cell lines. Although the determination of the expression levels was beneficial, RAD51 expression was not affected by either PTEN mutation or loss of PTEN expression (Figure 1A).

We generated a stable endometrial cancer cell line expressing wild-type PTEN (HEC-6 PTEN+) by introducing wild-type PTEN into a parental HEC-6 (PTEN null) cell line. The expression of exogenous PTEN was confirmed (Figure 1B) and the phosphorylation level of AKT (Ser-473) was decreased by PTEN introduction in HEC-6 PTEN + cells (Figure 1B), which suggests that the PI3K signaling is affected by wild-type PTEN. However, the expression of RAD51 was not increased by the introduction of PTEN (Figure 1B). Thus, RAD51 expression is indicated to be regulated in a PTEN-independent manner in endometrial cancers.

### Anti-proliferative effect of olaparib in endometrial cancer cell lines

**Figure 2:**
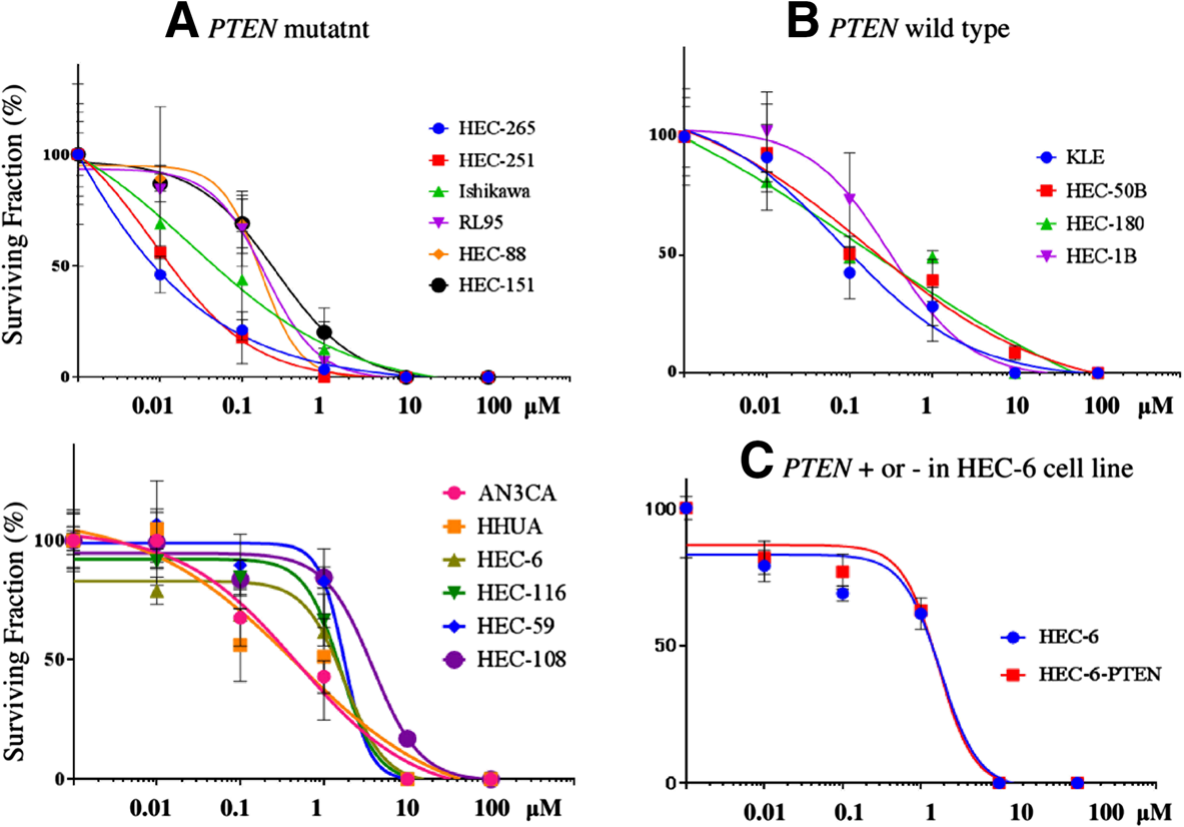
**Status of PTEN in endometrial cancer cells is irrelevant to the response to olaparib. (A) (B)** Each cell line was treated with 5 concentrations of olaparib, and the cell proliferation was evaluated by a clonogenic assay. Cells were cultured for 14–21 d. Cells were continuously exposed to olaparib with media during the incubation. All experiments were repeated 3 times, and each value is shown as the mean of 3 experiments ± SD. PTEN mutant cells (n = 12) are shown in (A: 6 cells in upper left and the other 6 in lower left), and wild-type cells (n = 4) are shown in **(B)**. **(C)** Clonogenic assay comparing HEC-6-PTEN + cells with parental HEC-6 cells. SF50 values were 1,800 nM in both cell lines.

We tested the anti-proliferative effects of olaparib in each of the 16 endometrial cancer cell lines by clonogenic assay under incubation of 14–21 days with continuous exposure to oraparib (Figure 2A and 2B). The SF50 values with olaparib varied from 8 nM to 2,500 nM (Table 2, Figure 2A and 2B). Four of the 16 (25%) cell lines demonstrated SF50 values at 100 nM or less, whereas 4 of the 16 (25%) cell lines exhibited SF50 values at 1,000 nM or more. Unexpectedly, the SF50 values were not significantly distinct between the PTEN mutant and PTEN wild-type cells (p = 0.26 by Student’s *t* test). Additionally, the lack of PTEN expression did not increase the sensitivity to olaparib (p = 0.27; Table 2). In the HEC-6 PTEN + cells, the SF50 value with olaparib was 1,500 nM, which was comparable with the SF50 value in the parental HEC-6 cells (Figure 2C).

**Table 2:** SF50 values to olaparib and the PTEN status in endometrial cancer cell lines

**PTEN**	**Cell lines**	**SF50 (nM)**	**Mean**	**SE**	**SD**	**P value (Student t test)**
Mutation	HEC-265	8	746	253	838	0.26
	HEC-251	15				
	Ishikawa	42				
	HEC-88	170				
	RL95-2	190				
	HEC-151	230				
	AN3CA	400				
	HHUA	400				
	HEC-6	1500				
	HEC-116	1600				
	HEC-59	1900				
	HEC-108	2500				
Wild type	KLE	100	215	49	85	
	HEC-180	200				
	HEC-50B	220				
	HEC-1B	340				
Expression (+)	HEC-265	8	809	305	915	0.27
	Ishikawa	42				
	RL95-2	190				
	HEC-1B	340				
	AN3CA	400				
	HHUA	400				
	HEC-6	1500				
	HEC-59	1900				
	HEC-108	2500				
Expression (-)	HEC-251	15	362	208	551	
	KLE	100				
	HEC-50	220				
	HEC-180	200				
	HEC-88	170				
	HEC-151	230				
	HEC-116	1600				

SE: Standard Error, SD: Standard Deviation.

We also examined the effect of PTEN in other cell lines. Knocking down PTEN by siRNA in two cell lines with wild-type PTEN (HEC-1B and HEC-50B) did not affect the sensitivity in clonogenic assay (Additional file 1: Figure S1A). In addition, introduction of wild-type PTEN plasmid (pcDNA3-HA-PTEN) into a PTEN mutant cell line (AN3CA) did not alter the sensitivity, compared with the control (Additional file 1: Figure S1B).

These results suggest that accumulated DSB, following PARP inhibition by olaparib, successfully induces anti-proliferative effects in specific endometrial cancer cell lines and that PTEN status is not a useful biomarker to predict the effectiveness.

### γH2AX and RAD51 foci formation following olaparib exposure in PTEN + and PTEN-null endometrial cells

**Figure 3:**
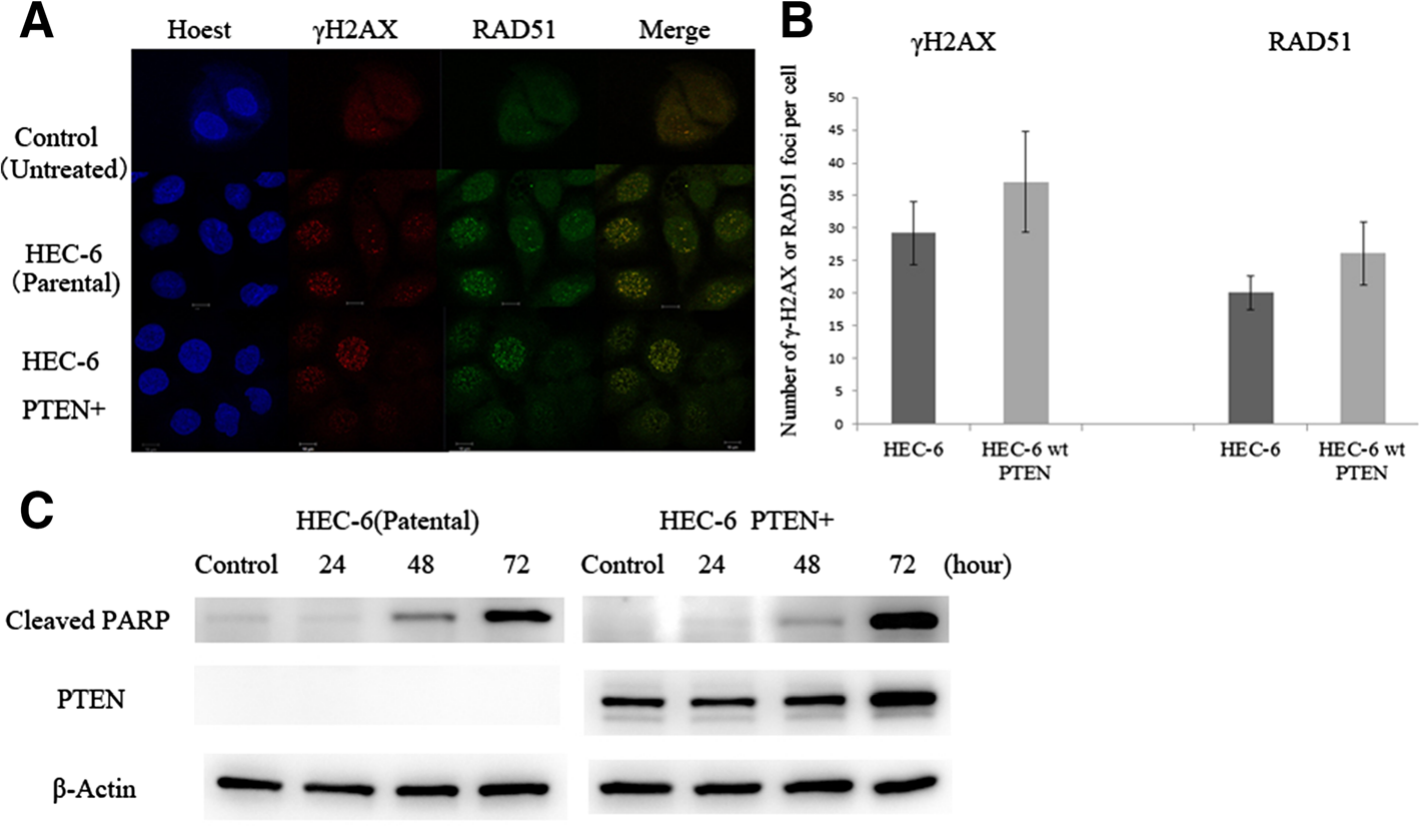
**γH2AX and RAD51 foci formation in HEC-6 cells after olaparib treatment. (A)** Immunofluorescence images of PTEN-/+ HEC6 cell lines: Hoechst-stained nuclei (blue), γ-H2AX (red), and RAD51 (green) after olaparib exposure (10 μM) for 24 h. **(B)** The number of γ-H2AX and RAD51 foci following exposure to olaparib (10 μM) was counted in the HEC-6 cell lines. The experiments were repeated 3 times, and each value is shown as the mean of 3 experiments ± SD. **(C)** Time course expression of cleaved PARP and PTEN in PTEN**-/+** HEC-6 cell lines. Proteins were extracted after 24 h of olaparib (10 μmol/L) exposure.

Nuclear foci of γH2AX and RAD51 were evaluated by immunofluorescence to observe responses to DNA damage in the parental HEC-6 and the HEC-6-PTEN + cells. The exposure to olaparib (10 μM) for 24 h induced foci formation of both γH2AX and RAD51 in the nuclei (Figure 3A). The number of γ-H2AX or RAD51 foci per cell was not significantly distinct between the parental HEC-6 and the HEC-6-PTEN + cells (Figure 3B). Since PARP cleavage is an important apoptosis marker, we evaluated whether the cleavage is distinct between the two cell lines. Following the exposure to olaparib (10 μM), cleaved-PARP was induced after 48 h and the induction level was increased after 72 h in the HEC-6 cells (Figure 3C). The level of cleaved-PARP was also comparable between the HEC-6 and HEC-6-PTEN + cells (Figure 3C). These data suggest that the expression of wild-type PTEN also does not affect apoptosis induction in HEC-6 cells.

### Sensitivity to IR was not affected by PTEN status in HEC-6 cells

**Figure 4:**
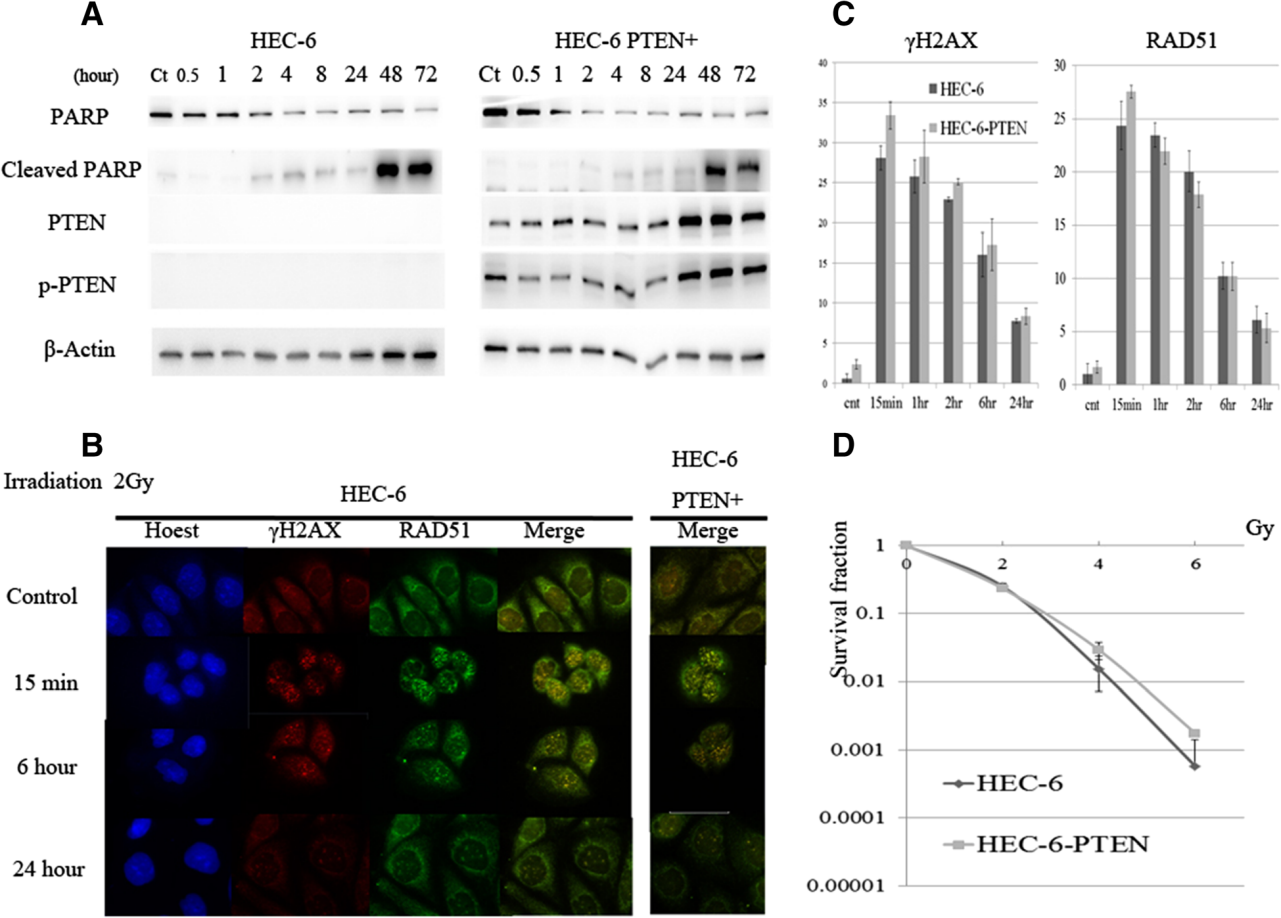
**Response to IR in HEC-6 PTEN + and parental HEC-6 cell lines. (A)** Total/cleaved PARP and total/phospho-PTEN expression in HEC-6 PTEN + and parental HEC-6 cell lines were examined by western blot analysis. Proteins were extracted after 10 Gy of IR at the indicated times. **(B)** Immunofluorescence images of PTEN-/+ HEC-6 cell lines: Hoechst-stained nuclei (blue), γ-H2AX (red), and RAD51 foci (green) after IR exposure (2 Gy). **(C)** The number of γ-H2AX and RAD51 foci following exposure to IR (2 Gy) was counted in the PTEN-/+ HEC-6 cell lines. The experiments were repeated 3 times, and each value is shown as the mean of 3 experiments ± SD. **(D)** Clonogenic assay in the PTEN-/+ HEC-6 cell lines after exposure to IR at the indicated doses (2–6 Gy).

Parental HEC-6 and HEC-6-PTEN + cells were exposed to IR at 10 Gy as a DNA damaging agent. The expression of PTEN, as well as the phosphorylation level of PTEN (Ser-380), was increased in HEC-6 PTEN + cells within 24 h following IR exposure (Figure 4A). Following the IR exposure, cleaved PARP was induced after 48 h, regardless of the presence of PTEN in these cells (Figure 4A). Using immunofluorescence, foci formation of γ-H2AX and RAD51 was observed following 2 Gy of IR exposure in both cell lines. The foci of these two proteins in the nuclei were induced within 15 min and continuously observed even after 6 h; the foci dispersed within 24 h (Figure 4B). The number of γ-H2AX and RAD51 foci was comparable between the parental HEC-6 cells and the HEC-6 PTEN + cells (Figure 4C). In addition, the clonogenic assay revealed that the cell survival fraction after IR exposure was not significantly distinct between the two cell lines at each dose tested (Figure 4D).

### Effects of olaparib and IR on the cell cycle of HEC-6 endometrial cancer cells

**Figure 5:**
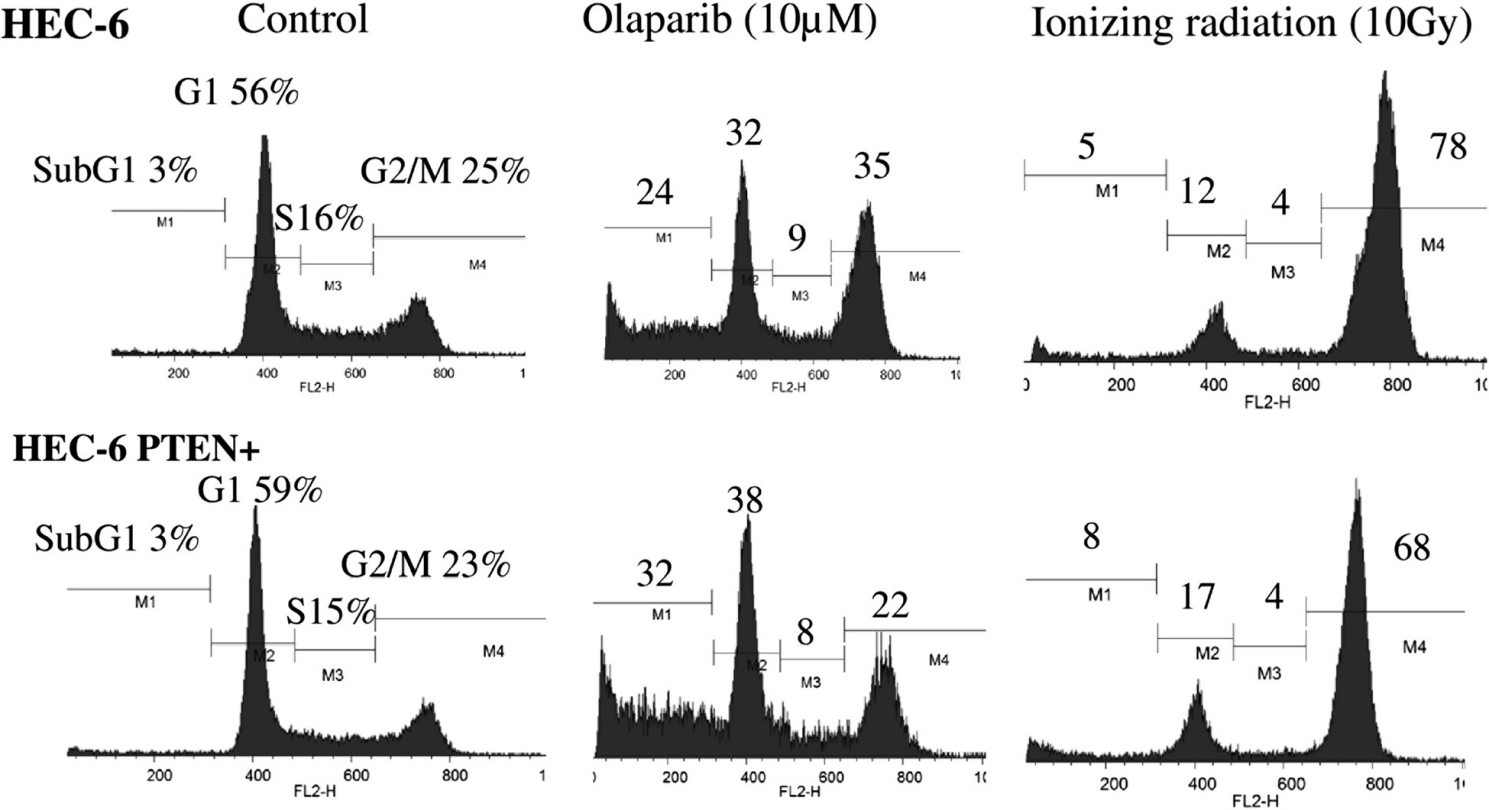
**Cell cycle population was not affected by PTEN status in the HEC-6 endometrial cancer cells.** Cell cycle populations following exposure to olaparib (10 μM, 72 h) or IR (10 Gy, 48 h) were determined by flow cytometry in PTEN-/+ HEC-6 cell lines.

Lastly, cell cycle analysis following exposure to olaparib (10 μM, 72 h) or IR (10 Gy, 48 h) was performed by flow cytometry in the HEC-6 and HEC-6-PTEN + cells. The sub-G1 population was markedly increased in both cell lines by either olaparib or IR (Figure 5). Although the population of G1, S, and G2/M phase was distinct between olaparib- and IR-treated cells, each population was comparable between the HEC-6 and HEC-6 PTEN + cells by either exposure. These data indicate that the cytotoxic and cytostatic effects of olaparib and IR were induced in a PTEN-independent manner in the HEC-6 cell lines.

## Discussion

Inhibiting PARP is a promising strategy in cancer cells, especially in cells with a deficiency in HR repair. Although BRCA1/2 play key roles in HR repair, the association of other tumor suppressor genes with HR repair is still debatable. In phase II clinical trials conducted in high-grade serous ovarian carcinomas, olaparib has been reported to be effective in certain patients without BRCA1/2 mutations [27,28]. The activity of olaparib (AZD2281, KU0059436) was demonstrated by Menear et al. [28]. The IC50 of olaparib (Compound 47 in the paper) was shown to be 6 nM and exposure to 100–300 nM olaparib inhibited PARP activity (quantified by a PAR formation) by 90–95% [6]. In addition, poly (ADP-ribose) expression was sufficiently suppressed by 1 μM or higher doses of olaparib, regardless of the SF50 values (ranging from 200 nM to 4,500 nM) [29]. Preclinical studies have suggested that PTEN deficiency causes HR defects and possibly induces sensitivity to PARP inhibitors [27]. Therefore, the inactivation of PTEN in cancer cells might be one of the possible mechanisms of HR defects independent from BRCA. In this study, using a panel of endometrial cell lines, we focused on (i) the anti-tumor effect of olaparib and its relationship with PTEN status, (ii) the association between PTEN and HR-related proteins (RAD51 and γ-H2AX), and (iii) the relationship between PTEN status and the response to IR exposure (another DNA damaging therapy).

Clonogenic assays revealed that sensitivity to olaparib is greatly distinct among endometrial cell lines, with SF50 values ranging from 8 nM to 2,500 nM. The high ratio (25%) of sensitive cells with SF50 values <100 nM suggests that endometrial cancers are good candidates for PARP inhibitors such as olaparib. Recently, the possibility of a relationship between PTEN status and sensitivity to PARP inhibitors has received much attention [3,22,30]. Dedes *et al*. reported that PTEN-deficient cells were more sensitive to a PARP inhibitor than PTEN-wild type endometrial cancer cells [31]. However, our results were not in agreement with their conclusion. In our study, the existence of *PTEN* mutations did not result in high sensitivity to olaparib. One of the 4 PTEN wild-type cells and three of the 12 PTEN mutant cells were classified as sensitive (SF50 ≦ 100 nM). Moreover, all the 4 resistant cell lines (SF50 > 1,000 nM) were PTEN mutant. The report by Dedes et al. included only 2 PTEN wild-type cell lines (HEC-1B and EFE-184), whereas 4 PTEN-wild type cell lines were included in our study. All the SF50 values in these 4 cell lines were 340 nM or lower. In addition, they did not include 3 of the 4 “resistant” (and PTEN mutant) cell lines in this study (HEC-6, HEC-116 and HEC-108) [31]. The contribution of PTEN inactivation was also negatively reported in prostate cancer cell lines (22RV1, DU145, and PC3) [22].

Therefore, we further examined the relationship between PTEN and HR-related proteins in endometrial cancer cells. RAD51 mediates the formation of DNA joints that link homologous DNA molecules [32]. Several recent reports have suggested that the loss of PTEN might be associated with the downregulation of RAD51 [19,33]. Our data suggest that RAD51 expression levels were not associated with the PTEN status in a panel of endometrial cells and that the introduction of PTEN does not upregulate RAD51 expression in HEC-6 cells. Dedes et al. reported that expression of RAD51 was not associated with the status of PTEN, and that RAD51 expression was predominantly observed in cytoplasm, not in nucleus [31]. These data are compatible with previous reports in astrocytes and prostate cancer cell lines [22,30] but not in agreement with reports in colorectal cancer cells [27]. Thus, the association of RAD51 and PTEN might be distinct among various types of tumors.

One of the earliest events in the signal transduction cascade initiating DSBs is the phosphorylation of serine 139 of histone H2AX (γH2AX) and RAD51 filament formation on DNA [34,35]. Our data showed that foci formations of γ-H2AX and RAD51 after olaparib exposure did not differ between parental HEC-6 and HEC-6-PTEN + cell lines. The data was also in agreement with the previous report that phospho-γ-H2AX foci formation by exposure to PARP inhibitor was not associated with PTEN status [31]. In previous reports, a PARP inhibitor induced G2/M arrest, which led to cell death [36,37]. In our study, olaparib induced both G2/M arrest and cleaved PARP expression in HEC-6 cell lines after 24 and 48 h of olaparib and IR exposure. It is crucial to note that these events are independent from PTEN status. Therefore, the response to olaparib-induced DNA damage is suggested to be independent from PTEN in endometrial cancer cells. Although PTEN deficiency was proposed as a predictive biomarker to PARP inhibition, our data with clear phenotypic change by olaparib but no impact of PTEN status suggests that PTEN is unlikely to be a predictive biomarker to PARP inhibitors in endometrial cancer.

We also examined whether the response to IR-induced DNA damage is affected by PTEN status. IR directly produces DNA DSBs [38]. After IR exposure, γH2AX and RAD51 foci formation was not significantly different between the parental HEC-6 cells and the HEC-6-PTEN + cells. Additionally, the cell cycle profile and cell proliferation were also not affected by exogenous PTEN when examined using flow cytometry and clonogenic assays. The response of RAD51 to IR exposure was similar in the endometrial cells without *PTEN* mutations.

Our study has some limitations. Predictive biomarkers for olaparib are still unknown. Further studies are warranted to elucidate whether PTEN inactivation is a biomarker for resistance to olaparib. The role of PTEN might be distinct when a PARP inhibitor was administered in combination with another drug (or irradiation), as the combination of a PARP inhibitor with cisplatin or irradiation was reported to be effective in PTEN deficient cells [39,40]. Additionally, the mechanism of RAD51 expression should be elucidated further.

## Conclusions

xIn conclusion, our findings have noteworthy implications: PTEN inactivation is not a good biomarker of olaparib treatment and PARP inhibitors might still provide a promising therapeutic strategy in certain endometrial carcinomas.

## Competing interests

All the authors declare no competing interests.

## Authors’ contributions

AM performed the experiments and wrote the manuscript. KO (corresponding author) supervised the experiments and wrote the manuscript. YI wrote the manuscript. OH-W, TK, TK, TF, KI, KS, YU, RK, KN, YM, TA, SN, TY, KK, YO, and TF contributed reagents, materials, experimental techniques, and data analysis. AE, NH, and KM contributed experiments using IR. HK established 11 HEC endometrial cancer cell lines. All authors read and approved the final manuscript.

## Pre-publication history

The pre-publication history for this paper can be accessed here:

http://www.biomedcentral.com/1471-2407/14/179/prepub

## Supplementary Material

Additional file 1: Figure S1 Response to olaparib was not influenced by PTEN expression in endometrial cancer cells. (A) PTEN was knocked down by siRNAspecific to PTEN at 10nM (si PTEN) in two PTEN-wild type cell lines (HEC-1B: left and HEC-50B: middle). Non-silencing siRNA (si CT) was used as a control. Parental cells are also used as a control (CT). Cells were continuously exposed to olaparib for 14 days of incubation with media. Suppression of PTEN expression was examined by Western blotting. In clonogenic assay, the surviving fraction was not significantly affected by knocking down PTEN in these two cell lines. (B) HA-tagged wild-type PTEN expression plasmid (pcDNA3-HA-PTEN) was transfected into PTEN-null AN3CA cells (ad PTEN). pcDNA3 plasmid with HA-tag alone (ad CT) was used as a control. Cells were continuously exposed to olaparib for 14 days of incubation with media. Exogenous PTEN expression was confirmed by Western blotting. In clonogenic assay, the surviving fraction was not significantly affected by introduction of wild-type PTEN in AN3CA cells. Click here for file

## References

[B1] Satoh MS Lindahl T Role of poly (ADP-ribose) formation in DNA repair Nature 1992 356 356 358 10.1038/356356a0 1549180

[B2] Tutt AN Lord CJ McCabe N Farmer H Turner N Martin NM Jackson SP Smith GC Ashworth A Exploiting the DNA repair defect in BRCA mutant cells in the design of new therapeutic strategies for cancer Cold Spring Harb Symp Quant Biol 2005 70 139 148 10.1101/sqb.2005.70.012 16869747

[B3] Dedes KJ Wilkerson PM Wetterskog D Weigelt B Ashworth A Reis-Filho JS Synthetic lethality of PARP inhibition in cancers lacking BRCA1 and BRCA2 mutations Cell Cycle 2011 10 1192 1199 10.4161/cc.10.8.15273 21487248 PMC3117132

[B4] Bryant HE Petermann E Schultz N Jemth AS Loseva O Issaeva N Johansson F Fernandez S McGlynn P Helleday T PARP is activated at stalled forks to mediate Mre11-dependent replication restart and recombination EMBO J 2009 28 2601 2615 10.1038/emboj.2009.206 19629035 PMC2738702

[B5] Yang YG Cortes U Patnaik S Jasin M Wang ZQ Ablation of PARP-1 does not interfere with the repair of DNA double-strand breaks, but compromises the reactivation of stalled replication forks Oncogene 2004 23 3872 3882 10.1038/sj.onc.1207491 15021907

[B6] Menear KA Adcock C Boulter R Cockcroft XL Copsey L Cranston A Dillon KJ Drzewiecki J Garman S Gomez S Javaid H Kerrigan F Knights C Lau A Loh VM Jr Matthews IT Moore S O'Connor MJ Smith GC Martin NM 4-[3-(4-cyclopropanecarbonylpiperazine-1-carbonyl)-4-fluorobenzyl]-2H-phthalazin- 1-one: a novel bioavailable inhibitor of poly (ADP-ribose) polymerase-1 J Med Chem 2008 51 6581 6591 10.1021/jm8001263 18800822

[B7] Fong PC Boss DS Yap TA Tutt A Wu P Mergui-Roelvink M Mortimer P Swaisland H Lau A O'Connor MJ Ashworth A Carmichael J Kaye SB Schellens JH de Bono JS Inhibition of poly (ADP-ribose) polymerase in tumors from BRCA mutation carriers N Engl J Med 2009 361 123 134 10.1056/NEJMoa0900212 19553641

[B8] Forster MD Dedes KJ Sandhu S Frentzas S Kristeleit R Ashworth A Poole CJ Weigelt B Kaye SB Molife LR Treatment with olaparib in a patient with PTEN-deficient endometrioid endometrial cancer Nat Rev Clin Oncol 2011 8 302 306 10.1038/nrclinonc.2011.42 21468130

[B9] Williamson CT Muzik H Turhan AG Zamo A O'Connor MJ Bebb DG Lees-Miller SP ATM deficiency sensitizes mantle cell lymphoma cells to poly (ADP-ribose) polymerase-1 inhibitors Mol Cancer Ther 2010 9 347 357 10.1158/1535-7163.MCT-09-0872 20124459 PMC3729269

[B10] Min A Im SA Yoon YK Song SH Nam HJ Hur HS Kim HP Lee KH Han SW Oh DY Kim TY O'Connor MJ Kim WH Bang YJ RAD51C-deficient cancer cells are highly sensitive to the PARP inhibitor olaparib Mol Cancer Ther 2013 12 865 877 10.1158/1535-7163.MCT-12-0950 23512992

[B11] Siegel R Naishadham D Jemal A Cancer statistics, 2012 CA Cancer J Clin 2012 62 10 29 10.3322/caac.20138 22237781

[B12] Oda K Stokoe D Taketani Y McCormick F High frequency of coexistent mutations of PIK3CA and PTEN genes in endometrial carcinoma Cancer Res 2005 65 10669 10673 10.1158/0008-5472.CAN-05-2620 16322209

[B13] Oda K Okada J Timmerman L Rodriguez-Viciana P Stokoe D Shoji K Taketani Y Kuramoto H Knight ZA Shokat KM McCormick F PIK3CA cooperates with other phosphatidylinositol 3’-kinase pathway mutations to effect oncogenic transformation Cancer Res 2008 68 8127 8136 10.1158/0008-5472.CAN-08-0755 18829572

[B14] Enomoto T Inoue M Perantoni AO Buzard GS Miki H Tanizawa O Rice JM K-ras activation in premalignant and malignant epithelial lesions of the human uterus Cancer Res 1991 51 5308 5314 1913654

[B15] Shoji K Oda K Nakagawa S Hosokawa S Nagae G Uehara Y Sone K Miyamoto Y Hiraike H Hiraike-Wada O Nei T Kawana K Kuramoto H Aburatani H Yano T Taketani Y The oncogenic mutation in the pleckstrin homology domain of AKT1 in endometrial carcinomas Br J Cancer 2009 101 145 148 10.1038/sj.bjc.6605109 19491896 PMC2713716

[B16] Salvesen HB MacDonald N Ryan A Jacobs IJ Lynch ED Akslen LA Das S PTEN methylation is associated with advanced stage and microsatellite instability in endometrial carcinoma Int J Cancer 2001 91 22 26 10.1002/1097-0215(20010101)91:1<22::AID-IJC1002>3.0.CO;2-S 11149415

[B17] Toda T Oku H Khaskhely NM Moromizato H Ono I Murata T Analysis of microsatellite instability and loss of heterozygosity in uterine endometrial adenocarcinoma Cancer Genet Cytogenet 2001 126 120 127 10.1016/S0165-4608(00)00400-3 11376804

[B18] Peiffer SL Herzog TJ Tribune DJ Mutch DG Gersell DJ Goodfellow PJ Allelic loss of sequences from the long arm of chromosome 10 and replication errors in endometrial cancers Cancer Res 1995 55 1922 1926 7728760

[B19] Shen WH Balajee AS Wang J Wu H Eng C Pandolfi PP Yin Y Essential role for nuclear PTEN in maintaining chromosomal integrity Cell 2007 128 157 170 10.1016/j.cell.2006.11.042 17218262

[B20] Puc J Keniry M Li HS Pandita TK Choudhury AD Memeo L Mansukhani M Murty VV Gaciong Z Meek SE Piwnica-Worms H Hibshoosh H Parsons R Lack of PTEN sequesters CHK1 and initiates genetic instability Cancer Cell 2005 7 193 204 10.1016/j.ccr.2005.01.009 15710331

[B21] Puc J Parsons R PTEN loss inhibits CHK1 to cause double stranded-DNA breaks in cells Cell Cycle 2005 4 927 929 10.4161/cc.4.7.1795 15970699

[B22] Fraser M Zhao H Luoto KR Lundin C Coackley C Chan N Joshua AM Bismar TA Evans A Helleday T Bristow RG PTEN deletion in prostate cancer cells does not associate with loss of RAD51 function: implications for radiotherapy and chemotherapy Clin Cancer Res 2012 18 1015 1027 10.1158/1078-0432.CCR-11-2189 22114138 PMC3378487

[B23] Kuramoto H Nishida M Morisawa T Hamano M Hata H Kato Y Ohno E Iida T Establishment and characterization of human endometrial cancer cell lines Ann N Y Acad Sci 1991 622 402 421 10.1111/j.1749-6632.1991.tb37884.x 2064198

[B24] Ikeda Y Oda K Nakagawa S Murayama-Hosokawa S Yamamoto S Ishikawa S Wang L Takazawa Y Maeda D Wada-Hiraike O Kawana K Fukayama M Aburatani H Yano T Kozuma S Taketani Y Genome-wide single nucleotide polymorphism arrays as a diagnostic tool in patients with synchronous endometrial and ovarian cancer Int J Gynecol Cancer 2012 22 725 731 10.1097/IGC.0b013e31824c6ea6 22635024

[B25] Shoji K Oda K Kashiyama T Ikeda Y Nakagawa S Sone K Miyamoto Y Hiraike H Tanikawa M Miyasaka A Koso T Matsumoto Y Wada-Hiraike O Kawana K Kuramoto H McCormick F Aburatani H Yano T Kozuma S Taketani Y Genotype-dependent efficacy of a dual PI3K/mTOR inhibitor, NVP-BEZ235, and an mTOR inhibitor, RAD001, in endometrial carcinomas PLoS One 2012 7 e37431 10.1371/journal.pone.0037431 22662154 PMC3360787

[B26] Tanikawa M Wada-Hiraike O Nakagawa S Shirane A Hiraike H Koyama S Miyamoto Y Sone K Tsuruga T Nagasaka K Matsumoto Y Ikeda Y Shoji K Oda K Fukuhara H Nakagawa K Kato S Yano T Taketani Y Multifunctional transcription factor TFII-I is an activator of BRCA1 function Br J Cancer 2011 104 1349 1355 10.1038/bjc.2011.75 21407215 PMC3078593

[B27] Banerjee S Kaye S PARP inhibitors in BRCA gene-mutated ovarian cancer and beyond Curr Oncol Rep 2011 13 442 449 10.1007/s11912-011-0193-9 21913063

[B28] Chen Y Zhang L Hao Q Olaparib: a promising PARP inhibitor in ovarian cancer therapy Arch Gynecol Obstet 2013 288 367 374 10.1007/s00404-013-2856-2 23619942

[B29] Chuang HC Kapuriya N Kulp SK Chen CS Shapiro CL Differential anti-proliferative activities of poly (ADP-ribose) polymerase (PARP) inhibitors in triple-negative breast cancer cells Breast Cancer Res Treat 2012 134 649 659 10.1007/s10549-012-2106-5 22678161 PMC4297209

[B30] McEllin B Camacho CV Mukherjee B Hahm B Tomimatsu N Bachoo RM Burma S PTEN loss compromises homologous recombination repair in astrocytes: implications for glioblastoma therapy with temozolomide or poly (ADP-ribose) polymerase inhibitors Cancer Res 2010 70 5457 5464 10.1158/0008-5472.CAN-09-4295 20530668 PMC2896430

[B31] Dedes KJ Wetterskog D Mendes-Pereira AM Natrajan R Lambros MB Geyer FC Vatcheva R Savage K Mackay A Lord CJ Ashworth A Reis-Filho J PTEN deficiency in endometrioid endometrial adenocarcinomas predicts sensitivity to PARP inhibitors Sci Transl Med 2010 2 53ra75 20944090 10.1126/scitranslmed.3001538

[B32] San Filippo J Sung P Klein H Mechanism of eukaryotic homologous recombination Annu Rev Biochem 2008 77 229 257 10.1146/annurev.biochem.77.061306.125255 18275380

[B33] Mendes-Pereira AM Martin SA Brough R McCarthy A Taylor JR Kim JS Waldman T Lord CJ Ashworth A Synthetic lethal targeting of PTEN mutant cells with PARP inhibitors EMBO Mol Med 2009 1 315 322 10.1002/emmm.200900041 20049735 PMC3378149

[B34] Rogakou EP Pilch DR Orr AH Ivanova VS Bonner WM DNA double-stranded breaks induce histone H2AX phosphorylation on serine 139 J Biol Chem 1998 273 5858 5868 10.1074/jbc.273.10.5858 9488723

[B35] Sung P Krejci L Van Komen S Sehorn MG Rad51 recombinase and recombination mediators J Biol Chem 2003 278 42729 42732 10.1074/jbc.R300027200 12912992

[B36] McCabe N Turner NC Lord CJ Kluzek K Bialkowska A Swift S Giavara S O'Connor MJ Tutt AN Zdzienicka MZ Smith GC Ashworth A Deficiency in the repair of DNA damage by homologous recombination and sensitivity to poly (ADP-ribose) polymerase inhibition Cancer Res 2006 66 8109 8115 10.1158/0008-5472.CAN-06-0140 16912188

[B37] Farmer H McCabe N Lord CJ Tutt AN Johnson DA Richardson TB Santarosa M Dillon KJ Hickson I Knights C Martin NM Jackson SP Smith GC Ashworth A Targeting the DNA repair defect in BRCA mutant cells as a therapeutic strategy Nature 2005 434 917 921 10.1038/nature03445 15829967

[B38] Ward JF The yield of DNA double-strand breaks produced intracellularly by ionizing radiation: a review Int J Radiat Biol 1990 57 1141 1150 10.1080/09553009014551251 1971840

[B39] Minami D Takigawa N Takeda H Takata M Ochi N Ichihara E Hisamoto A Hotta K Tanimoto M Kiura K Synergistic effect of olaparib with combination of cisplatin on PTEN-deficient lung cancer cells Mol Cancer Res 2013 11 140 148 10.1158/1541-7786.MCR-12-0401 23239809

[B40] Chatterjee P Choudhary GS Sharma A Singh K Heston WD Ciezki J Klein EA Almasan A PARP inhibition sensitizes to low dose-rate radiation TMPRSS2-ERG fusion gene-expressing and PTEN-deficient prostate cancer cells PLoS One 2013 8 e60408 10.1371/journal.pone.0060408 23565244 PMC3614551

